# A Female Case of X-Linked Retinoschisis with Macular Hole Bilaterally

**DOI:** 10.1155/2020/8824995

**Published:** 2020-08-15

**Authors:** A. Altun

**Affiliations:** Department of Ophthalmology, Bahcesehir University, Faculty of Medicine, Istanbul, Turkey

## Abstract

**Purpose:**

We aimed at reminding that X-linked retinoschisis may also be seen in female patients and share our vitreoretinal surgical experience.

**Methods:**

The patient underwent pars plana vitrectomy including the closure of the macular holes with inverted ILM flap technique bilaterally. Lens extractions were performed by phacoemulsification during the removal of silicone oil endotamponade. *Patient*. An 18-year-old girl with X-linked retinoschisis and large macular holes in both eyes presented to the clinic of ophthalmology. It was confirmed that the patient had RS1 mutation

**Results:**

Nine-month-follow-up was uneventful for retinal findings. Significant improvement in visual acuity was achieved, and macular holes were remained closed.

**Conclusion:**

In cases with large macular holes due to XLR, an inverted ILM flap technique might be safe and effective. Four-month-silicone-endotamponade might be sufficient.

## 1. Introduction

X-linked retinoschisis (XLR) is a rare macular disease that is observed in hemizygous males and homozygous females. XLR is a common cause of macular dysfunction in young men due to the allelic heterogeneity at the retinoschisis (X-linked, juvenile) locus 1 (RS1), with a prevalence of 1 in 5000 to 1 in 25,000 [1, 2]. Today, it is estimated to be very low compared to men, but the prevalence of XLR in the female population has not yet been determined. In this paper, we aimed at sharing our findings and the results of vitreoretinal surgery in an 18-year-old girl with bilateral macular hole with XLR.

## 2. Case Presentation

An 18-year-old female patient was admitted to our clinic with a decrease in visual acuity for 3 years. Intravitreal antivascular endothelial growth factor (anti-VEGF) and dexamethasone implant injections were applied 3 times to the right eye and 4 times to the left eye due to the suspicion of macular edema in different clinics. In the detailed anamnesis, the patient reported that she could see very well in childhood and did not use glasses and there was no history of trauma. There was a consanguineous marriage between mother and father (they were the children of each other's aunts).

**Figure 1:**
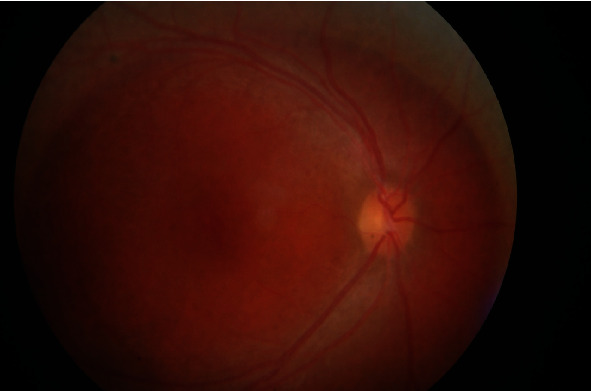
Fundus photo of the right eye.

**Figure 2:**
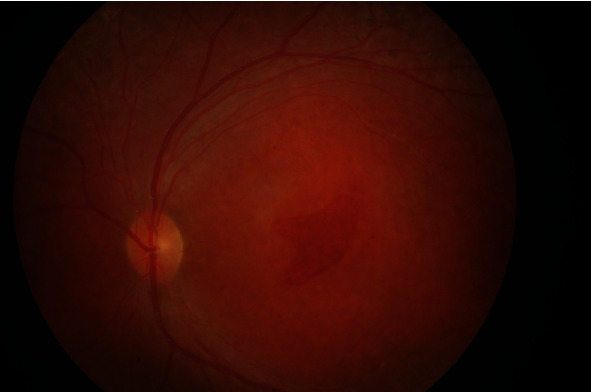
Fundus photo of the left eye.

**Figure 3:**
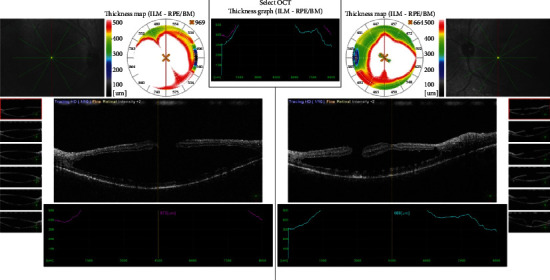
Preoperative optical coherence image of the eyes.

In the ophthalmological examination, it was determined that the patient was orthophoric and eye movements were free in all directions. The patient had no refractive error. The best-corrected visual acuity (BCVA) was at the level of counting fingers from 2 meters in the right eye and 3 meters in the left eye. The cornea, anterior chamber, iris, and lens findings were within normal limits. Intraocular pressure (IOP) was 12 mm in the right eye and 13 mmHg in the left eye. In funduscopic examination, macular hole (Figures 1 and 2) was detected in both eyes, which was wider in the left eye. Optical coherence tomography (OCT) revealed macular holes as well as the presence of retinoschisis in both eyes (Figure 3). There were also spots on the retina of the patient in the peripheral region. Electroretinogram showed a decrease in b-wave amplitude bilaterally. The patient did not have any additional problems at night vision. The patient was diagnosed as X-associated retinoschisis (XLR) due to funduscopic, OCT, and ERG findings.

## 3. Methods

After obtaining the informed consent, under general anesthesia, 23 gauge pars plana vitrectomy, inner limiting membrane (ILM) peeling, and the closure of macular holes were performed with an inverted ILM flap technique.

During PPV, a third-hand effect was created with the help of perfluorocarbon to stabilize the macula and prevent iatrogenic tearing during the ILM peeling phase. At the end of the operation, 1000 centistokes silicone oil endotamponade was implanted in both eyes, and the patient was kept in the prone position for 7 days in the postoperative period. Silicone endotamponade was removed after 4 months in both eyes. During the removal of silicone oil, cataract surgeries were performed with the phacoemulsification technique for lens opacification that developed post-PPV.

## 4. Results

**Figure 4:**
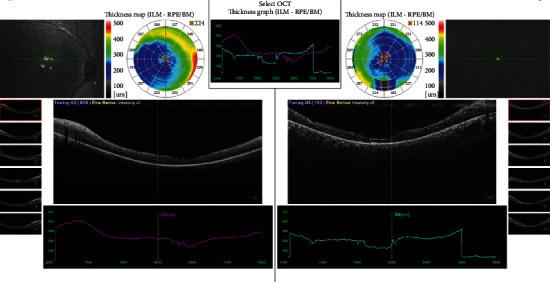
Postoperative optical coherence image of the eyes.

During the postoperative follow-up period, no drug was needed for IOP control and always remained within normal limits. Within a month, BCVA improved to 2/10 in the right eye and 3/10 in the left eye. The patient was confirmed to have genetically RS1 mutation. During follow-up, the maculas were flat and the holes were closed. Three months later, the posterior subcapsular cataract developed in both eyes. During the removal of silicone oil in the fourth month after PPV, the patient's cataract surgeries were performed in the same sessions. At the last visit of the patient (at the 9^th^ month after PPV), BCVA improved to 4/10 in the right eye and 6/10 in the left eye, there was no macular hole, and retina was attached in both eyes (Figure 4).

## 5. Discussion

XLR is one of the rare retinal diseases whose genetic cause is not yet fully understood. The association of XLR with the RS1 mutation has been previously reported in the literature [1, 2]. Our case had also RS1 mutation. Most of the cases reported in the literature have male gender [1–6]. One of the conditions that make our case special is her gender. The reason for this may be the close relative relationship between mother and father. When her detailed story was questioned, it was learned that the patient's two aunts had similar problems, but unfortunately, we did not have the chance to reach these people and confirm their eye findings.

Bilateral retinoschisis is the most frequently observed finding, as in our case. The diagnosis of XLR is made by funduscopic and OCT findings and excluding other possible causes. In XLR, retinoschisis usually affects the macula bilaterally, but only one case has been reported in the literature where only the peripheral retina is affected unilaterally [7]. In the majority of cases reported to have XLR, the macular hole is unilateral. In our case, there was a bilateral macular hole, as in the case presented by Gautam et al. [8].

Today, the pathogenesis of XLR is not fully understood [2]. Gass [3] suggested that “Muller cell cone” was a key factor in the pathogenesis of congenital XLR. The Muller cell cone is a focal point for foveal division and cyst formation in both pathologies. In XLR, the accumulation of defective retinoschisin protein in and around Muller cells causes cystic voids in multiple retinal layers. The combination of these cysts leads to the formation of larger cysts, resulting in larger glandular cavities. In the uppermost region of the schisis cavities, degeneration of the retina takes place because of tangential traction caused by elevation of the retinal tissue. Breakage of inner layers occurs and a macular hole forms.

Methods such as intravitreal dexamethasone [9] or gene therapy [10] in XLR treatment have been described in the literature. Before presenting our clinic, the patient had been injected with intravitreal anti-VEGF or dexamethasone implant in different clinics. Today, there is no evidence that anti-VEGF drugs are effective in macular edema or retinal dissociation that may develop in XLR.

We preferred a surgical treatment option for our case. We performed PPV and an inverted ILM flap technique to close the macular holes. Although the macular holes were closed in one month, we extended the silicone oil endotamponade for up to 4 months to support the separated retinal layers. During PPV, we observed that the cortical vitreous was strictly connected to the retina in both eyes. This experience reinforces the traction hypothesis of Shukla et al., Greven et al., and Shanmugam et al. [4–6] on the macula in pathophysiology. Yu et al. [11] reported that vitreoretinal traction associated with interface tight connection was found in 9% of their cases.

In the majority of cases reported to have XLR, the macular hole is unilateral. In our case, there were bilateral macular hole, as in the case presented by Yu et al. [11].

## 6. Conclusion

X-linked retinoschisis (XLR) can rarely be observed in females, especially in daughters of close-relative parents. Inverted ILM flap application under perfluorocarbon can be a safe technique in the surgical treatment of large macular holes in patients with XLR. In these cases, it may be sufficient for the silicone oil to remain in the eye for 4 months for endotamponade.

## Data Availability

Not Required.

## Additional Points

*Summary Statement*. In rhegmatogenous X-linked retinoschisis cases, the inverted ILM flap technique and four-month silicone tamponade might be a sufficient and efficient treatment method.
